# What to expect from drug targeting factor XI?

**DOI:** 10.1093/cvr/cvac091

**Published:** 2022-06-22

**Authors:** Magdolna Nagy, Hugo ten Cate

**Affiliations:** Department of Biochemistry, Cardiovascular Research Institute Maastricht (CARIM), Maastricht University, Maastricht, the Netherlands; Department of Biochemistry, Cardiovascular Research Institute Maastricht (CARIM), Maastricht University, Maastricht, the Netherlands; Department of Internal Medicine, Maastricht University Medical Center+, Maastricht, the Netherlands; Thrombosis Expertise Center, Heart+ Vascular Center, Maastricht University Medical Center, Maastricht, the Netherlands; Center for Thrombosis and Hemostasis (CTH), University Medical Center of the Johannes Gutenberg University Mainz, Mainz, Germany

**Keywords:** Factor XI, Thrombosis, Anticoagulants, Prevention, Pleiotropy

With the landmark studies showing comparable efficacy and safety for direct oral anticoagulants (DOACs) and vitamin K antagonists (VKAs), the principle of antithrombotic therapy through targeting single coagulation proteases was demonstrated and clinically embraced.^1^ DOACs are rapidly replacing VKA for many common indications, like atrial fibrillation (AF) and venous thromboembolism (VTE). Where traditional anticoagulants, either VKA or (low molecular weight; LMW) heparins target the synthesis of multiple proteases (VKA) or inhibit various proteases indirectly, through the cofactor antithrombin (heparin/LMWH) respectively, DOACs inhibit one single protease, factor Xa, or thrombin. Interfering with (specific) coagulation proteases not only reduces the risk of thrombosis but may also have several off target effects that merit consideration.^2^

The clinical efficacy of DOACs inspired the development of other targeted anticoagulants, also aimed at coagulation proteases, including factor (F) XI(a). FXI can be activated via either of two enzymes, FXIIa in the contact activation pathway and thrombin, generated by the downstream intrinsic coagulation pathway. Thus, FXI is positioned at a crossroad of two key components of the coagulation cascade: contact system and intrinsic pathway (*Figure 1*).

**Figure 1:**
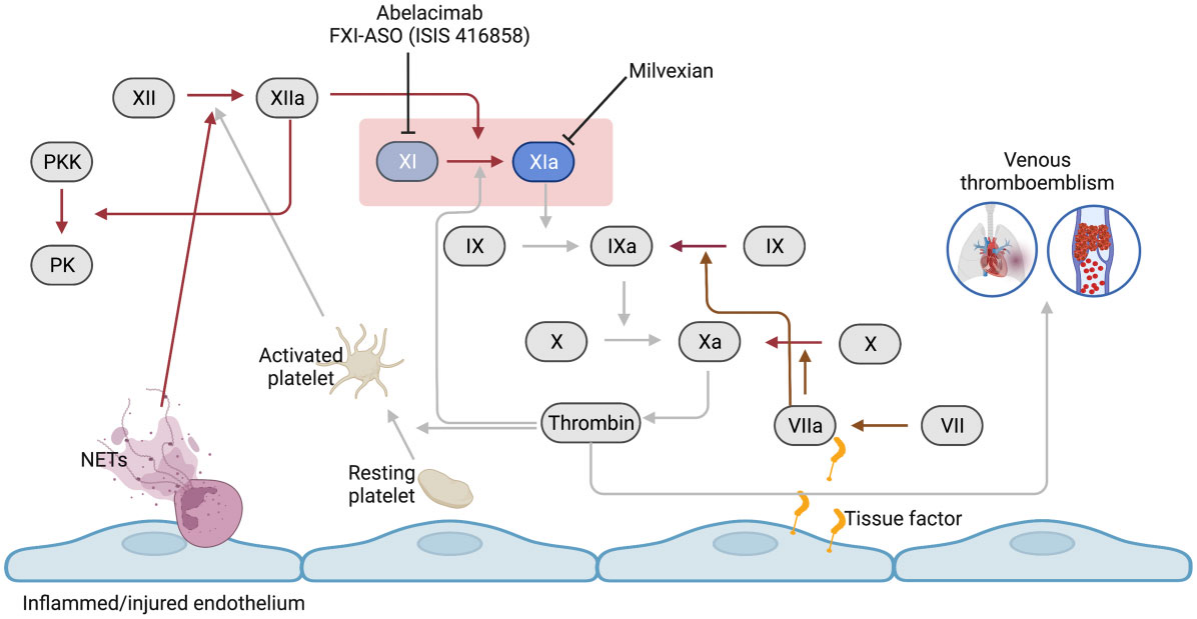
Central role of FXI(a) in the coagulation system. Inflamed or damaged endothelial layer results in exposure of tissue factor and activation of platelets and neutrophils leading to activation of the extrinsic pathway (via FVII) or contact pathway (via FXII), respectively. FXI activated by either by FXIIa or the feedback loop of thrombin will result in further thrombin generation though the intrinsic pathway. Attenuation of FXI activity inhibiting either FXI or FXIa leads to reduced thrombin formation and venous thromboemblism (light arrows), while the extrinsic pathway and the contact pathway remain unaffected (dark arrows).

In vivo, FXIa activates the intrinsic coagulation cascade, and the enzyme is inactivated through binding to one of the available protease inhibitors, in particular C1-inhibitor (±70%), followed by alpha-2 antiplasmin (10%), alpha 1 antitrypsin (10%), and antithrombin (±10%); under inflammatory conditions FXIa may also be captured by plasminogen activator inhibitor 1 (reviewed in^3^). By monitoring such enzyme-inhibitor complexes in vivo, the involvement of FXI activation was recently shown in patients with acute VTE, where levels of FXIa-C1-inhibitor and FXIa-alpha-1-antitrypsin were linked to risk of recurrent VTE.^4^ Previous studies also revealed elevated levels of FXIa and enzymes from the contact pathway in subjects with arterial thromboembolic disorders.^3^

By drug targeting FXI(a), it was deemed likely that effective inhibition of the intrinsic route, ie thrombin and fibrin formation, would also yield effective thrombosis prevention. However, why would one target yet another serine protease if DOAC already are so effective? The main reason is that it is expected that inhibiting FXI(a) may have less impact on hemostasis than downstream anticoagulants including DOACs. This assumption is based on the clinical observation that deficiencies of any of the contact factors (FXII, high molecular weight kininogen, prekallikrein) do not yield a bleeding diathesis, while deficiency in FXI results in a variable but much less severe bleeding diathesis than any deficiency in the downstream intrinsic system, including factors VIII or IX (hemophilia A and B).^5^

Clinical trials have now provided sufficient proof of concept for the efficacy of FXI inhibition; in the first trial, silencing FXI gene expression lowered FXI levels and dose dependently prevented postoperative VTE in patients undergoing elective knee replacement surgery.^6^ A second trial explored a monoclonal antibody (abelacimab) against FXI in the same knee arthroplasty setting and showed dose dependent superior efficacy compared to enoxaparin, at similar low bleeding rates.^7^ In the recent ‘Milvexian for the Prevention of Venous Thromboembolism’ trial, the same human ‘model of VTE’ was applied to study the antithrombotic efficacy of this oral, small molecule inhibitor of FXIa.^8^ Milvexian dose dependently reduced the rate of postoperative VTE: 7 of 28 (25%) patients taking 25 mg, in 30 of 127 (24%) taking 50 mg, and in 8 of 123 (7%) taking 200 mg of milvexian, as compared to 54 of 252 patients (21%) taking enoxaparin, the standard comparator LMWH agent. Major bleeding or clinically relevant non-major bleeding occurred in 1% and 2% for milvexian and enoxaparin, respectively. The data thus suggest that further gain in antithrombotic protection is indeed possible without concurrent dose dependent increments in major bleeding risk. While these data stem optimistic about further improving the benefit risk ratio of anticoagulants, studies in patients at greater baseline risk of bleeding are needed to judge this safety profile outside the rather strictly organized setting of elective knee surgery. Important indications to consider are the risks of unprovoked bleeding in elderly subjects with additional risk factors for bleeding like anaemia, renal failure, recent ischaemic stroke, history of major bleeding and also in those following complex surgery. Given that FXI deficiency comes with a highly variable bleeding risk, mostly associated with trauma/surgery, the safety profile of anti-FXI(a) agents needs to be much more firmly established before one can conclude that gain in safety is within reach. While such studies are ongoing, proposals for reversal of factor XI(a) inhibitors with antifibrinolytic compounds or recombinant factor VIIa have already been published.^5^

## What else can be expected from FXIa inhibition?

Importantly, FXI inhibition will reduce FXa and thrombin generation too, so the impact on downstream coagulation will to some extend also include similar antithrombotic, but also off target effects. Like for direct inhibitors of FXa and thrombin such off target effects may relate to ‘vascular protective’ properties because of protease activated receptor (PAR) cell signalling modification,^2^ and possibly other complex diseases like diabetic nephropathy, fibrosis and cancer through PAR modulation as well. Furthermore, FXIa may have additional specific effects to consider. Besides the main substrate FIX, FXIa may also activate other coagulation factors including FV, FVIII, and FX (reviewed in^3^). Tissue factor pathway inhibitor (TFPI) can also be proteolytically cleaved by FXIa, which may paradoxically increase the potency of the tissue factor route to generate thrombin^3^; conceptually, this may limit both anticoagulant efficacy and support hemostasis. A profibrinolytic effect of FXIa inhibition was shown in a rabbit thrombosis model, possibly due to reduced thrombin mediated inhibition of thrombin activatable fibrinolytic inhibitor.^3^ The latter effect might explain the increased potency of dose dependent FXIa inhibition, as compared to enoxaparin, but it may also contribute to the mild bleeding pattern in FXI deficient subjects.

Several lines of evidence show that FXIa may link with inflammation. Prochemerin can be cleaved by contact activation generated FXIa to an intermediate that is subject to cleavage by plasma carboxypeptidases to yield chemerin, an adipokine, and chemoattractant. Animal studies show that FXI deficiency modifies inflammation^9^ through contact system and cytokine regulated mechanisms. Recent data from Pallares *et al.*^10^ based on a proteomics analysis of plasma from patients with acute VTE shows that among 444 proteins investigated, a substantial number were associated with FXI:c, including proteins from immune pathways linked to thrombo-inflammation, extracellular matrix interaction, lipid metabolism, and apoptosis.^10^ Additional interactions of FXI in oxidative stress mechanisms and interactions with polyanions including the glycosaminoglycan heparin are discussed in the study by Pallares *et al.*^10^.

The development of inhibitors targeting FXI(a) is a next element in the antithrombotic medication array, with demonstrated activity against VTE at acceptable bleeding rates. While the efficacy:safety ratio of such agents in more complex patient settings, including those at risk of myocardial infarction and stroke, is under investigation, an open eye should be kept at any off-target effects that such selective agents may have in particular on thrombo-inflammatory mechanisms.

### Funding

M.N. is a young talent fellow from the Contrast consortium, sponsored by the Netherlands Heart Foundation. She also received postdoctoral support from the REG-MED XS consortium.

**Conflict of interest:** H.t.C. has received financial compensation for research or consultations, from Bayer, Pfizer, Leo, Alveron, Viatris, Astra Zeneca, Portola, Alexion, Galapagos. M.N. reports no additional conflict of interest.

## Authors

**Biography:** Dr. Magdolna Nagy graduated as Clinical Laboratory Scientist from the University of Debrecen in Hungary and received her PhD from Maastricht University in the Netherlands. Her PhD focused on discovering novel pathways in thrombus formation with particular emphasis on platelet (dys)function and platelet-leukocyte crosstalk under the supervision of Prof. Johan Heemskerk, Prof. Steve Watson, and Dr. Judith Cosemans. After obtaining her PhD, she joined the group of Prof. Hugo ten Cate and Dr. Henri Spronk as a postdoctoral researcher at Maastricht University to study the impact of (anti)coagulation on cardiac regenerative therapy aiming to restore the heart function ex vivo. In 2019, she received a Contrast Young Investigator Voucher from the NHS supported CONTRAST Consortium to investigate the role of contact activation in ischaemia reperfusion injury in acute ischaemic stroke. Her current research is focusing on unravelling the impact of contact activation pathway in venous thromboembolism and acute ischaemic stroke and finding novel coagulation biomarkers at the same time. Her dedication to translational research aids to bring these novel coagulation biomarkers towards clinics.

**Biography:** Hugo ten Cate Graduated in medicine at the University of Amsterdam in 1987 and obtained a PhD degree based on an academic thesis on the Clinical and experimental studies with a LMWH, at the University of Amsterdam in 1987. Between 1988 and1990 he was a postdoctoral fellow at the laboratory of prof Robert D Rosenberg and dr. Kenneth A Bauer, Beth Israel Hospital and Harvard Medical School Boston where he studied mechanisms of thrombosis using novel coagulation activation markers. Next, he completed training in Internal medicine at Slotervaart hospital and Academic Medical Center Amsterdam, combining it with translational research at Sanquin (prof Erik Hack) and at the Academic Medical Center (laboratory of Experimental Internal medicine, prof Pieter Reitsma). After being awarded a Clinical Established Investigatorship from the Dutch Heart Foundation he joined the Maastricht University Medical Center (MUMC+) and Cardiovascular Research Institute (CARIM) in 2002 as a Principal investigator and Professor in Internal medicine, particularly in Clinical Thrombosis and Haemostasis. He is the Director of the Maastricht Thrombosis Expertise Center, at the Heart and Vascular Center, MUMC+, a staff member of the division of Vascular Medicine, dept. of Internal medicine and Division leader Blood and board member CARIM. Between 2012 and 2016 he was a Fellow of the Gutenberg Research College, Gutenberg University and since 2017 Adjunct-professor, Center for Thrombosis and Haemostasis (CTH), Gutenberg University Medical Center, Mainz, Germany. His main lines of research center around mechanisms of venous and arterial thrombosis, which he explores in translational research within several (inter)national consortia. He published over 450 articles.
